# Development of Water Repellent, Non-Friable Tannin-Furanic-Fatty Acids Biofoams

**DOI:** 10.3390/polym14225025

**Published:** 2022-11-19

**Authors:** Elham Azadeh, Ummi Hani Abdullah, Nurul Basirah Md Ali, Antonio Pizzi, Christine Gerardin-Charbonnier, Philippe Gerardin, Wan Sarah Samiun, Siti Efliza Ashari

**Affiliations:** 1 Faculty of Science and Technology, LERMAB, University of Lorraine, Bd. des Aiguillettes, BP 70239, 54506 Vandoeuvre Les Nancy, France; 2 Department of Wood and Fiber Industries, Faculty of Forestry and Environment, Universiti Putra Malaysia, Serdang 43400, Selangor, Malaysia; 3 Institute of Tropical Forestry and Forest Products (INTROP), Universiti Putra Malaysia, Serdang 43400, Selangor, Malaysia; 4 LERMAB-ENSTIB, University of Lorraine, 27 Rue Philippe Seguin, 88000 Epinal, France; 5 Department of Chemistry, Faculty of Science, Universiti Putra Malaysia, Serdang 43400, Selangor, Malaysia

**Keywords:** tannin-furanic foams, esterified tannins, friability, water repellence, surface free energy, FTIR, MALDI-TOF

## Abstract

Tannin-furanic foams were prepared with a good yield using the addition of relatively small proportions of a polyflavonoid tannin extract esterified with either palmitic acid, oleic acid, or lauric acid by its reaction with palmitoyl chloride, oleyl chloride, or lauryl chloride. FTIR analysis allowed us to ascertain the esterification of the tannin, and MALDI-TOF analysis allowed us to identify a number of multi-esterified flavonoid oligomers as well as some linked to residual carbohydrates related to the equally esterified tannin. These foams presented a markedly decreased surface friability or no friability at all, and at densities lower than the standard foam they were compared to. Equally, these experimental foams presented a much-improved water repellence, as indicated by their initial wetting angle, its small variation over time, and its stabilization at a high wetting angle value, while the wetting angle of the standard foam control went to zero very rapidly. This conclusion was supported by the calculation of the total surface energy of their surfaces as well as of their dispersive and polar components.

## 1. Introduction

Tannins are renewable, natural materials that are readily available. They can be extracted from the bark and wood of certain trees and from agricultural waste. They have been traditionally extracted industrially for more than a century for use in the manufacture of heavy leathers. Their use has been extended in the last decades to several other industrial applications. They are widely used to manufacture wood adhesives [1,2,3], but extensive research on their utilization has led to a variety of other possible applications, such as metal primers, pharmaceuticals, and many other uses [4,5]. Among these, one of the most interesting applications is for biobased foams for thermal and acoustic insulation.

Tannin-based foams have been prepared from renewable, natural raw materials to substitute for petrochemical resources [6,7]. Tannin-based foams are one example of biobased foams, composed of 95% relatively inexpensive natural raw materials that are considered less- or nontoxic. Condensed tannin and hydrolysable tannin are two different types of tannin possessing different structures. The structure of hydrolysable tannins is gallic and ellagic acid-based, taking the form of chains of pentagalloyl glucose and of their degradation products, such as castalagin and castalin. Some authors have stated that they are not recommended for producing foams due to their fragility and friability [8,9]. However, recent work on hydrolysable tannin-based non-isocyanate polyhydroxy urethane foams has shown these conclusions to be incorrect [10,11].

Polyflavonoid tannin extract from the bark of the black mimosa (*Acacia mearnsii*, formerly *mollissima*, de Wildt) is one of the two most common commercial condensed tannins [12]. Such condensed tannins consist of 2–10 flavonoid units, which are reactively responsive towards aldehydes [1,13,14]. Tannin-based foams, or more exactly tannin-furanic-based foams, are prepared by the reaction of tannin with furfuryl alcohol with or without aldehydes, mainly formaldehyde [6], although recent research has succeeded in preparing them using other aldehydes [15,16,17] or even with no aldehydes at all [18]. Formaldehyde reacts with both tannins’ phenolic aromatic ring sites [1] as well as with the furanic ring of the furfuryl alcohol cross-linking them [19]. These foams can be produced at room temperature without any pre-polymerization. These materials can compete with synthetic, industrial phenol-formaldehyde foams [13,20,21,22].

Notwithstanding their excellent fire resistance imparted by both the tannin and the furanic moieties, some drawbacks still do persist for these foams. One of the most annoying observed and reported drawbacks is their surface friability and their over-absorption of water when used as thermal insulating material. An initial indication that both such drawbacks could possibly be solved was the coupling of small amounts of fatty acid-esterified tannins to the tannin constituting the majority of the foam [23]. Esterification of a number of different flavonoids and of flavonoid tannins is known in the literature in a variety of application fields and using a number of different approaches. Thus, in the medical pharmaceutical field enzymatic esterification of model flavonoid compounds with unsaturated fatty acids has been reported [24,25], where enzymatic conversion showed a better percentage at between 70% and 85% yield with different flavonoids with oleic acid and linoleic acid than transesterification for linoleic acid [24]. Conversely, esterification of glycosylated model flavonoid compounds was showed by NMR to occur mainly on the glucosides linked to the flavonoids [26]. More recently, a study on tannin esterification for use in materials using anhydrides was investigated as a route to synthesize tannin esters possessing varying ester chain length and degree of substitution (DS) to study the impact on polylactic acid comparing it with other systems of flavonoid esterification [27]. These various routes to tannin ester formation included (i) with acetic anhydride [28]; (ii) with acyl chloride with stearoyl chloride [29]; and (iii) transesterification with vinyl laurate [30]. These routes indicated the favoured flavonoid sites where esterification occurred, namely on all the phenolic and C3 alcoholic sites by anhydride, on the flavonoid B-ring phenolic –Ohs by a transesterification approach, and mainly on the phenolic –OH groups of both the B- and A-rings by reaction with an acyl chloride. This latter is also confirmed by two other studies on model compounds such as catechin, naringenin, and mesquitol, where the phenolic hydroxyls were esterified with ease [31,32]. It is however strange that an alcoholic –OH such as the one on the flavonoid C3 is esterified with an anhydride and not found with an acyl chloride, as it does not really make sense.

With these premises, the work presented here is a more in-depth investigation of how to solve such drawbacks in the best and most effective way, to determine the control parameters to achieve this, and finally to eliminate such drawbacks.

## 2. Materials and Methods

### 2.1. Materials

Spray-dried mimosa tannin (*Acacia mearnsii* formerly *mollissima*, de Wildt) extract powder was supplied by Silva Chimica (St. Michele Mondovi’, Italy). Furfuryl alcohol and lauroyl chloride were purchased from Acros Organics (Geel, Belgium). Para-toluene-sulphonic acid (pTSA), diethyl ether, acetaldehyde, and glutaraldehyde were purchased from Sigma-Aldrich (St. Louis, MO, USA). Formaldehyde was provided by Merck-Schuchardt OHG (Hohenbrunn, Germany). Oleoyl chloride and palmitoyl chloride were acquired from Alfa Aesar (Kandal, Germany). Chloroform was obtained from Merck (Darmstadt, Germany).

### 2.2. Synthesis of Oil-Grafted Tannin

The method to synthesize the oil-grafted tannin was modified from that reported by Rangel et al. [23] to improve the preparation of the oil-grafted tannin. To begin, 5 g of commercial mimosa tannin extract was weighed and transferred into a 250 mL round bottom flask. Then, 5 g of palmitoyl chloride was added, followed by 80 mL of chloroform. At room temperature, the reaction mixture was magnetically agitated for at least 12 h and then evaporated until dry under reduced pressure, while maintaining the temperature below 40 °C. Finally, a dark brown solid of oil-grafted tannin was obtained. The same procedure outlined above for palmitoyl chloride was used for both oleyl chloride and lauroyl chloride (maintaining the same molar ratio addition of fatty acid) to examine the effect of different fatty acid chains on synthesis percentage yield.

### 2.3. Testing and Characterization of Oil-Grafted Tannin Extract

#### 2.3.1. Fourier Transform Infrared (FTIR) Spectroscopy Analysis

The FTIR spectra were collected on a Perkin Elmer Spectrum One (Villebon-sur-Yvette, France) supplied with an ATR-FTIR unit. A few milligrams of each oil-grafted tannin sample were placed on a diamond/ZnSe crystal sample holder. The spectra were acquired by accumulation of 10 scans at a resolution of 4 cm^−1^ and in the 650–4000 cm^−1^ wavelength range. The results obtained were recorded and examined using Spectrum software (Perkin Elmer, Waltham, MA, USA). A natural tannin extract spectrum was used as control.

#### 2.3.2. Matrix-Assisted Laser Desorption Ionization–Time-of-Flight (MALDI-TOF) Analysis

Water/acetone (50/50 volume) was used to dissolve the oil-grafted tannin (4 mg/mL), and the solutions were added to the matrix solution of 10 mg/mL in acetone. 2,5-dihydroxy benzoic acid was used as the matrix to ease placing the sample on the sample-holder plate. Red phosphorus was used for instrument calibration (LaserBio Labs, Valbonne, France). A concentrated solution of 10 mg/mL of sodium chloride (NaCl) in distilled water was mixed into the matrix to enhance ion formation. The sample was added to the matrix solution and was divided into three parts. From 0.5 to 1 µL of three parts of the sample solution and one part of the NaCl solution was put on the MALDI target. The MALDI target was placed into the spectrometer after solvent evaporation. The peaks in the spectrum can present themselves at the actual molecular weight of the chemical species or increased by 23 Da, this being the molecular weight of the Na+ linked to the molecule from the NaCl added to the matrix to facilitate the flight of heavier oligomers. Sometimes both forms of the same species, with and without Na+, can be present in the same spectrum. The MALDI-TOF spectra were recorded on an AXIMA Performance instrument (Shimadzu Scientific Instruments, Manchester, UK). The irradiation source was a pulsed nitrogen laser with 3 ns intervals at a wavelength of 337 nm. The measurements were carried out using the following conditions: polarity, positive; flight path, linear; mass, high (20-kV accelerating voltage); and 100 to 150 pulses per spectrum. The delayed extraction technique was used to apply delay times of 200 to 800 ns, and the ion gate was set at 400 Da. The spectra are exact at ±1 Da.

### 2.4. Foam Preparation

Mimosa tannin extract was thoroughly mixed by hand in a beaker with water, furfuryl alcohol, and formaldehyde (37% solution). Oil-grafted mimosa tannin was added into the mixture according to the formulations in Table 1. Then, para-toluene-sulfonic acid (pTSA) catalyst as a 65% solution in water and diethyl ether (blowing agent) were added to the mixture, followed by 20 s of vigorous stirring. Within 2–3 min of the addition of the catalyst and blowing agent, a black foam was obtained. The foam was left for at least 24 h to allow evaporation and eliminate the residual blowing agent trapped in it. An equivalent tannin foam without the addition of oil-grafted mimosa tannin was also prepared as a control.

### 2.5. Characterization of Tannin-Based Foam

#### 2.5.1. Density

After fully drying the samples, the bulk density, ρ, was calculated as the weight/volume ratio of each sample of a standard dimension of 30 mm × 30 mm × 30 mm. The density of each sample was recorded.

#### 2.5.2. Friability

Evaluation of friability was conducted manually according to Rangel et al. [23]. The samples were rubbed without stress or adding pressure 5 times against the wipe tissue paper surface along a 3.5 cm distance. The foam with the added oil-grafted tannin was rubbed at the same time as the standard foam against the wipe tissue paper to provide comparison. The comparison was based on visual evaluation of the amount of foam residue that was left on the paper after rubbing. The friability was assessed manually [23,33] in accordance with the following protocol: Each sample was rubbed five times, without additional pressure or stress, against the paper surface along a 3.5 cm distance. Kimtech Science paper was used (Precision Wipes Tissue Wipers, Kimberly-Clark Professional, Nanterre, France). For each formulation, the hydrophobized foam and the standard one were rubbed simultaneously on the paper to afford comparison to the control experiment.

#### 2.5.3. Contact Angle Measurement

The contact angle measurements were determined according to Martha et al. [34]. The determination of the contact angle of water to measure the wettability of the tannin foam was performed with a video measuring system from Krüss Gmb (Villebon-sur-Yvettes, France), equipped with a high-resolution CCD camera. The tannin foams were placed on the top of a table in front of the CCD camera. A water droplet of 20 microlitre was placed onto the foam surface using a micropipette to always obtain the same size droplet. The drop shapes on the surface of the tannin foam were captured by the CCD camera and measured for a period of 180 s. Image J 1.46 software with DropSnake plugin analysis was used to measure the contact angle for both the left and the right side of the individual image of the droplet. The values obtained were then averaged. In total, 19 images were taken for each recorded drop to obtain a curve of contact angle versus wetting time.

Surface free energy (SFE) calculations were determined using Young’s equation [35]. Young’s equation states that:

(1) $$\gamma_{s}=\gamma_{sl}+\gamma_{l}\cos\theta$$

where $\gamma_{s}$, $\gamma_{sl},$ and $\gamma_{l}$ are regarded as the SFE of a solid, the SFE of the solid–liquid interface, and the SFE of the liquid, respectively, and *θ* is the value of the contact angle between the solid and the measuring liquid. However, the value of *γ_s_* and *γ_sl_* cannot be obtained directly from Young’s equation and using another equation by Dupré is needed [36]. Dupré introduced the concept of work adhesion between phase a and phase b by the following equation:

(2) $$W_{ab}=\gamma_{a}+\gamma_{b}-\gamma_{ab}$$

By assuming “*a*” is a solid and “*b*” is a liquid, Equation (3), the Young–Dupré [36] equation, will be obtained by exchanging Equation (1) to Equation (2):

(3) $$W_{sl}=\gamma_{l}\left(1+\cos\theta \right)$$

The work of cohesion of a solid and liquid can be defined as

(4) $$W_{ss}=\gamma_{s}+\gamma_{s}-0=2\gamma_{s}$$

(5) $$W_{ll}=\gamma_{l}+\gamma_{l}-0=2\gamma_{l}$$

According to Berthelot’s assumption, the adhesion work between the solid and liquid *Wsl* is equal to the geometric mean of the cohesion work of a solid and liquid individually:

(6) Wsl=WssWll12

The basis for the surface energy calculation, Equation (7), was obtained by combining all the equations from Equations (3)–(6):

(7) Wsl=2γsγl12=γl1+cosθ

Many other methods have been introduced to identify the amount of SFE of a sample. One of the approaches that can be used was by Owens–Wendt–Rabel–Kaelble [37,38,39], where total surface free energy is the combination of polar and dispersive parts:

(8) $$\gamma_{l}=\gamma_{l}^{d}+\gamma_{l}^{p}$$

(9) $$\gamma_{s}=\gamma_{s}^{d}+\gamma_{s}^{p}$$

where $\gamma_{l}^{d}$ and $\gamma_{s}^{d}$ are represented as the dispersive surface energy for liquid and solid, respectively. Meanwhile, $\gamma_{l}^{p}$ and $\gamma_{s}^{p}$ are the polar surface energy components of the liquid and solid, respectively.

## 3. Results and Discussion

### 3.1. FTIR Results of Oil-Grafted Tannin

**Figure 1:**
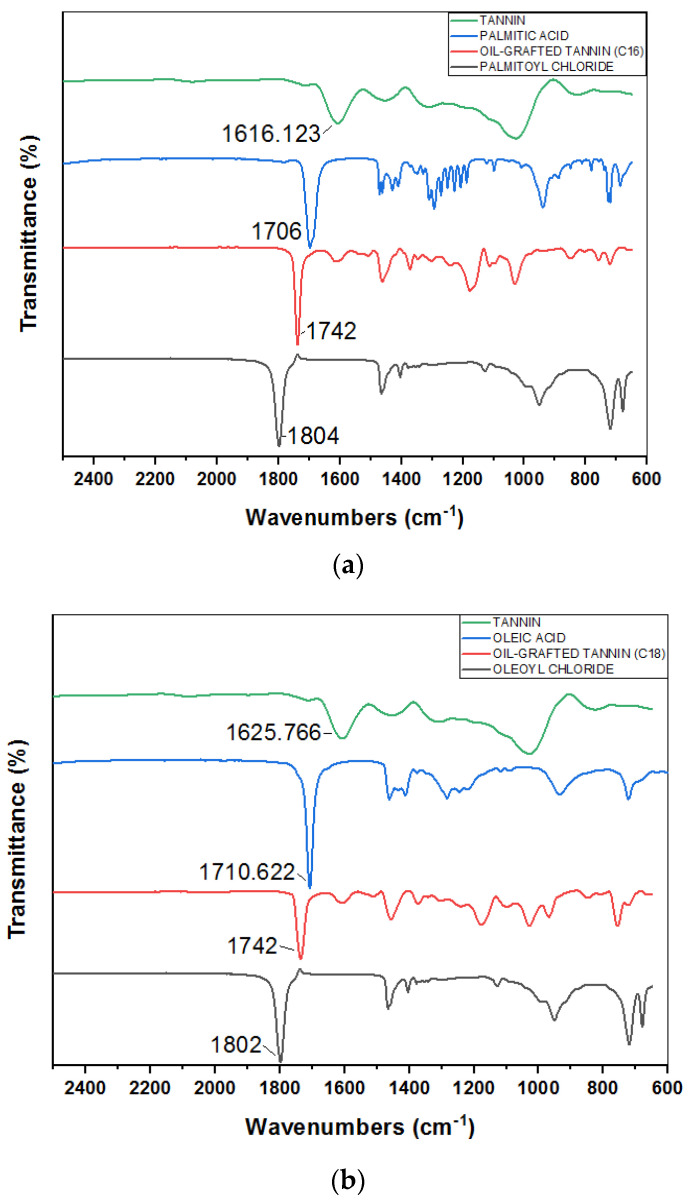

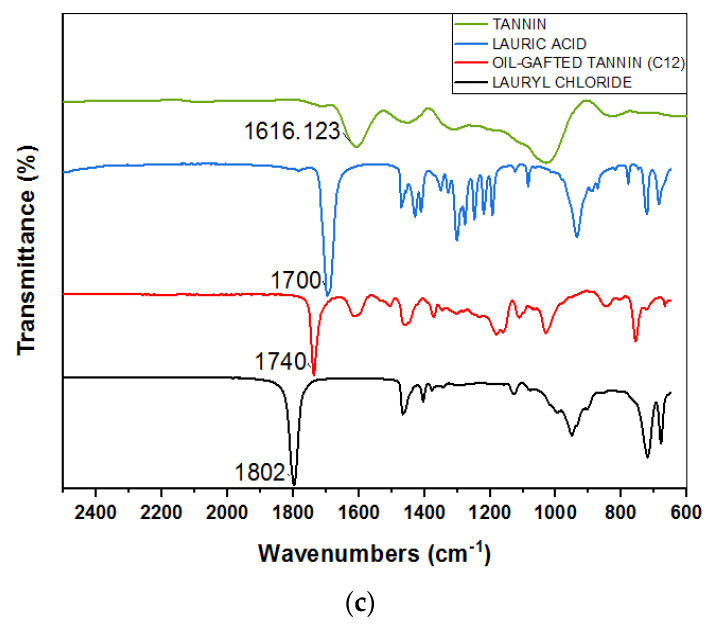
(**a**). FTIR spectra of mimosa tannin, palmitic acid, oil-grafted tannin using palmitoyl chloride, and palmitoyl chloride. (**b**). Comparison of FTIR spectra of mimosa tannin, oleic acid, oil-grafted tannin using oleoyl chloride, and oleoyl chloride. (**c**). Comparison of FTIR spectra of mimosa tannin, lauric acid, oil-grafted tannin using lauryl chloride, and lauryl chloride.

Figure 1a shows the FTIR spectra of mimosa tannin, palmitic acid, palmitoyl chloride, and oil-grafted tannin using palmitoyl chloride. In the FTIR spectra of the oil-grafted tannin, a sharp peak appeared at 1742 cm^−1^, proving the presence of ester groups in the oil-grafted tannin. The spectrum for palmitoyl chloride shows that a sharp peak at 1804 cm^−1^ is due to the presence of the C=O stretch band for acid chlorides [40]. This sharp peak disappears when using palmitoyl chloride to synthesize oil-grafted mimosa tannin. The absorption band at 1616.12 cm^−1^ in the tannin spectrum is attributed to the C–H vibration in the aromatic ring of the mimosa tannin [41,42]. A sharp peak appearing at 1706 cm^−1^ in the palmitic acid spectrum is assigned to the C=O stretch band for the carboxylic acid group [40]. Figure 1b shows the FTIR spectra of mimosa tannin, palmitic acid, palmitoyl chloride, and oil-grafted tannin using palmitoyl chloride [40,41,42,43,44]. Figure 1c shows the FTIR spectra of mimosa tannin, lauric acid, lauroyl chloride, and oil-grafted tannin using lauroyl chloride [40,41,42,43,44]. It can be concluded that different fatty acid chloride chains did not influence the FTIR result but that they affected the yield of oil-grafted tannin, as shown in Table 2.

**Figure 2:**
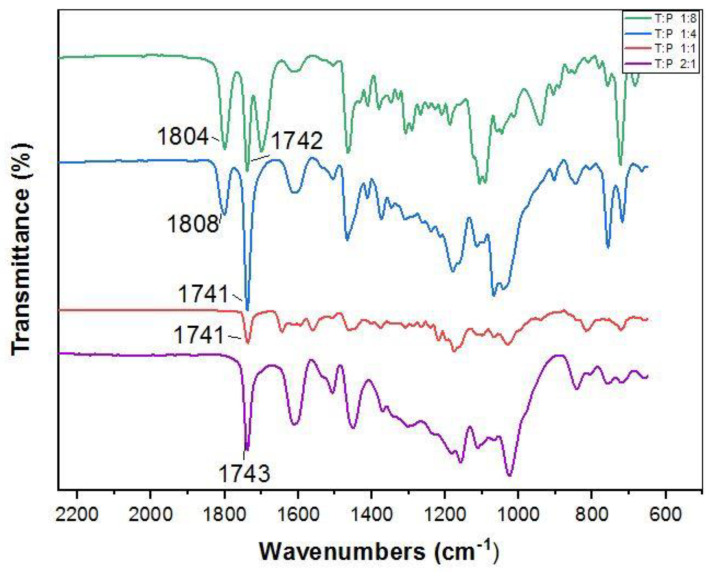
Comparison of different compositions to produce oil-grafted tannin using palmitoyl chloride.

Oil-grafted tannins using different mass ratios of fatty acid chlorides and tannin were also prepared. As different fatty acid chloride chains did not influence the FTIR result, only palmitoyl chloride was consequently maintained for further investigation. Table 3 shows different compositions used to synthesize the oil-grafted tannin by using palmitoyl chloride [40,44]. Based on Figure 2, an absorption band appears at 1804 cm^−1^ and 1808 cm^−1^ in the spectra of the oil-grafted tannins using palmitoyl chloride with mass ratios of 1:8 and 1:4, respectively. This peak is due to the presence of an excess of the acid chloride group of palmitoyl chloride in the oil-grafted tannin [40]. Contradictorily, the spectra for mass ratios of 1:1 and 2:1 show that there was no presence of peaks from the acid chloride group but only an ester group existed. Therefore, the 1:1 mass ratio was used to prepare the oil-grafted tannin.

### 3.2. MALDI-TOF Analysis

#### 3.2.1. MALDI-TOF Analysis of Oil-Grafted Tannins from Palmitoyl Chloride

**Figure 3:**
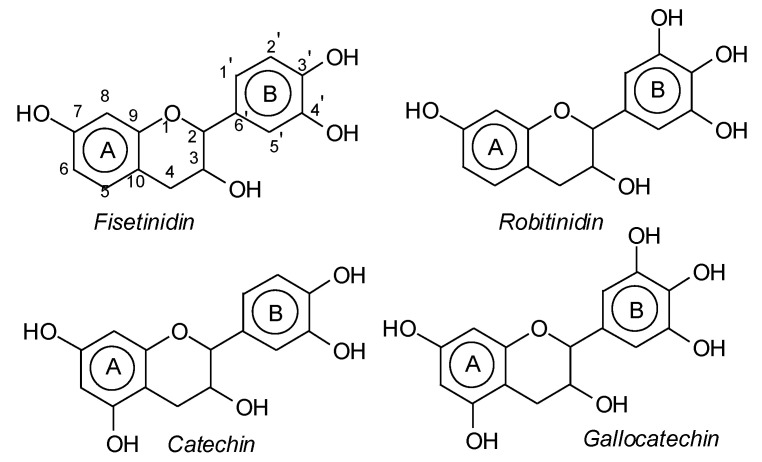
The structure of the four flavonoid types constituting mimosa tannin are shown. A- and B-rings are indicated and the site numbers have been indicated on the fisetinidin structure.

Mimosa tannin is a natural polymer that consists of various types of polyphenolic oligomers. As the commercial tannin extract was used, there were also some residual carbohydrates present, generally fragments of hemicelluloses [1]. Basically, the formation of the oligomers in the condensed tannin consists of four types of flavonoid units, which are fisetinidin (A), robinetinidin and catechin (B), and gallocatechin (C), with respective masses of 274.3 Da, 290.3 Da, and 306.3 Da [1]. They are linked to each other mainly at C4-C6 but also C4-C8 (Figure 3). It must be pointed out that robinetinidin and catechin have the same molecular mass, but in mimosa tannin robinetinidin constitutes 70% of the flavonoids present while catechin constitutes only approximately 5% [1] (Figure 3). It must be kept in mind however that whenever robinetinidin is mentioned in an oligomer it could also be catechin. These four flavonoid units are shown in Figure 3.

**Figure 4:**
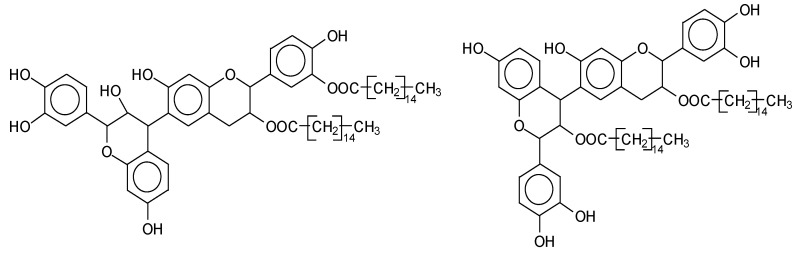
Two of the possible isomer structures for the (fisetinidin)_2_-(palmitate)_2_ oligomer, showing that the palmitoyl residue can have reacted both on the C3 alcoholic –OH and on one of the phenolic –OHs.

MALDI-TOF analysis of condensed flavonoid tannins is a proven and established technique [45,46,47,48,49]. Table 4 shows the characterization of oil-grafted tannin with palmitoyl chloride by MALDI-TOF. Fisetinidin-palmitate (calculated at 515 Da) is protonated and without Na+, indicating that the palmitate residues can be on any of the tannin –OHs. Nonetheless, it is most likely to be on the C3, the OH of which has a more alcoholic character. However, it is at least likely that many isomers of each flavonoid-monopalmitate do occur, with the C3-substituted one possibly predominating, as shown by the structures proposed for the species at 515 Da, 529 Da, 747 Da, 782 Da, and 807 Da (Table S1). This is also valid for all the higher oligomers identified and shown in Table S1, with the exception of diesterified flavonoid monomers, triesterified flavonoid monomers, and dimers such as the species at 747 Da, 1027 Da, 1281 Da, 1297 Da, 1315 Da, and 1331 Da (Table S1). In these, at least one of the palmitate residues has reacted on one of the phenolic –OHs of the flavonoids and is thus linked to a phenolic aromatic ring. Robinetinidin-palmitate is protonated without a Na+ (calculated 529 Da). The same site of reaction is valid for the diprotonated gallocatechin-palmitate, which appears at 545 Da without Na+. The species fisetinidin-(palmitate)_2_ without Na^+^ (calculated 747 Da) is the first showing that both the alcoholic –OH on the flavonoid C3 and one of the phenolic’s –OHs are also esterified without being able to define if the site esterified on the aromatic ring is on the flavonoid B-ring –OHs or A-ring OHs. The compound (fisetinidin)_2_-(palmitate)_2_, without and with Na^+^ at 782 Da and 807 Da, indicates that the second palmitate residue on this flavonoid dimer is more probably or more abundantly on the C3 of the second flavonoid unit, but reaction with one of the phenolic groups (although in a minority) cannot be excluded (Figure 4).

**Figure 5:**
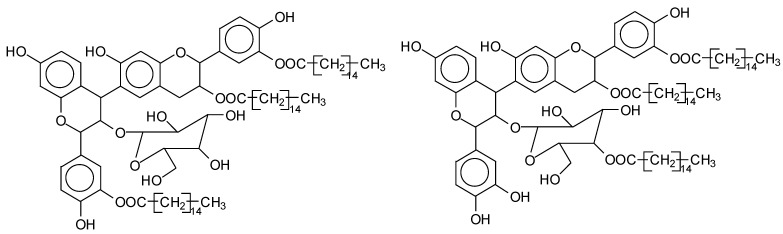
Example of the structures of two possible isomers of the identified species glucose-(fisetinidin)_2_-(palmitate)_3_ at 1442 Da.

**Figure 6:**
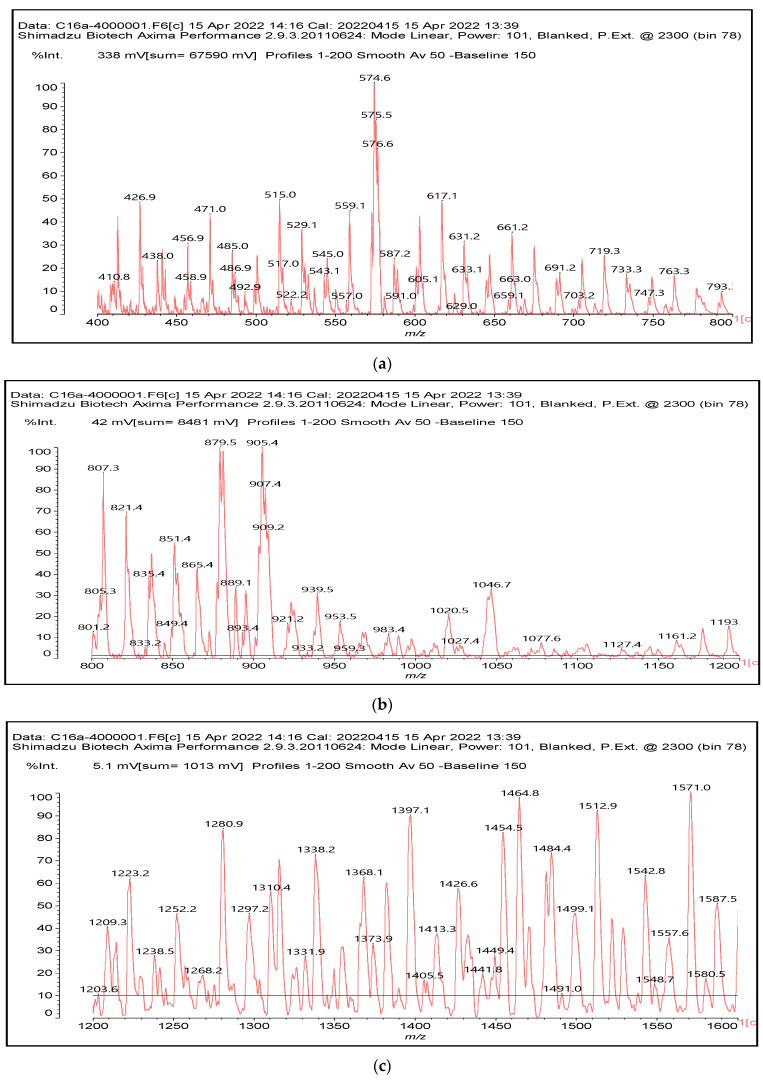
MALDI-TOF mass spectra of oil-grafted tannin with palmitoyl chloride (**a**) from 400 Da to 800 Da, (**b**) from 800 Da to 1200 Da, (**c**) from 1200 Da to 1600 Da.

This demonstrates that flavonoid oligomers are also esterified. The compound (gallocatechin)_2_-(palmitate)_4_- without Na+ indicates that at least two or more (Figure 5) of the phenolic –OHs do esterify, not just the two alcoholic –OHs in C3s. Figure 6a–c shows a MALDI-TOF analysis of modified oil-grafted mimosa tannin extract using palmitoyl chloride with the molecular weight ranging from 400 to 1600 Da. One unusual but indicative species formed that is worth noting is the one at 1442 Da, where a triesterified flavonoid dimer is linked to a glucose residue. It is well-known that in flavonoid tannin extracts there are residual carbohydrates and fragments of hemicelluloses, some of which even link to flavonoid moieties [46,47]. This is the case of the species at 1442 Da, but considering the high proportion of –OH groups on glucose, one cannot say if the palmitoyl residues are linked just to the flavonoids or if some are linked onto the carbohydrate (Figure 5). Two of the possible isomer structures are shown in Figure 5, but other isomers where the distribution of the palmitoyl residues can be different could equally be present (Figure 6).

#### 3.2.2. MALDI-TOF Analysis of Oil-Grafted Tannins from Oleoyl Chloride

**Figure 7:**
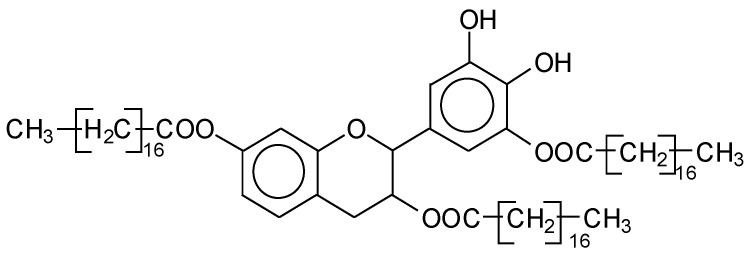
Examples of the structure of a robinetinidin-(oleate)_3_, with Na+.

Table S1 and Figure S1a–d (Supplementary Material) show the characterization of oil-grafted tannin with oleoyl chloride using MALDI-TOF. The compound fisetinidin-oleate monoester is protonated with Na+ (calculated 561 Da), indicating that the oleate residues can be on any of the –OHs, but they are most likely to have reacted with the -OH on the C3 that has a more alcoholic character. The same is true for the robinetinidin-oleate monoester, normal and deprotonated with Na+ (calculated 575–577 Da). The fisetinidin-(oleate)_2_ diester at 801 Da is the first compound that demonstrates that both the alcoholic –OH on C3 and one of the phenolic –OHs are also esterified, without being able to define if it is with the B-ring or A-ring –OHs. The same can be said for the series of compounds in Table S2 at 841 Da, 1105 Da, 1331 Da, 1355 Da, 1609 Da, and 1630 Da. Of these the species at 1105 Da, a robinetinidin trioleate, must be particularly noted (Figure 7), indicating that a flavonoid can be extensively esterified on the majority of its –OH groups.

At 1609 and 1630 Da is a robinetinidin pentaoleate ester that has all the –OHs of the flavonoid esterified, indicating that phenolic –OHs do react with the fatty acid with ease, notwithstanding their slightly acid behaviour (Table S1). Their several flavonoid dimers esterified, such as the ones at 1149 Da, 1331 Da, 1355 Da, 1338 Da, 1500 Da, 1601 Da, 1609 Da, 1630 Da, and 1688 Da, which shows that higher flavonoid oligomers are also esterified with ease. As for the flavonoid palmitates, a species at 1500 Da is an oleate triester linked to a flavonoid dimer to which, as in the palmitate esters’ case, is also linked a glucose residue of the hemicelluloses present in a small amount in the extract. In principle, the pattern of compounds formed is very similar to what was observed for the flavonoid palmitate esters. Figure S2a–d show the MALDI-TOF spectra of modified oil-grafted tannins using oleoyl chloride with the molecular weight ranging from 400 to 2000 Da.

#### 3.2.3. MALDI-TOF Analysis of Oil-Grafted Tannins from Lauryl Chloride

Table S2 and Figure S2 (Supplementary Material) show the characterization of oil-grafted tannins with lauryl chloride using MALDI-TOF mass spectrometry. The chemical species found for the esterification of tannin with lauryl chloride are rather similar to what was already observed in Table 4 and Table 5 for tannin esterified with palmitoyl chloride and oleyl chloride, but series of oligomers are more and more clearly visible in the spectrum, probably due to the lower molecular weight of the laurate residue and its possibly higher mobility. Thus, there are four clear peak series, each separated by a repeating motive of 182 Da, that are clearly identifiable in this particular case. The first of these is formed by the peaks at 1011 Da, 1193 Da, 1375 Da, 1557 Da, 1740 Da, 1922 Da, 2104 Da, 2286 Da, and 2468 Da. These are formed by the progressive addition of one lauryl residue at a time on a glucose-(robinetinidin)_2_-fisetinidin oligomer at 1011 Da, starting with the glucose-(robinetinidin)_2_-fisetinidin-laurate (no Na+) oligomer at 1193 Da and ending with the glucose-(robinetinidin)_2_-fisetinidin-(laurate)_8_ (no Na+) oligomer at 2468 Da. As inferred, the esterification of a carbohydrate side chain linked to the flavonoid also occurs extensively with the tannin laurate (Table S2) (Supplementary material).

The second clearly identifiable series of peaks, also separated by a repeating mass of 182 Da, is the one formed by the peaks at 633 Da, 815 Da, 997 Da, 1179(s) Da, 1361(s) Da, 1543 Da, 1724 Da, 1906 Da, 2089 Da, and 2271 Da. These are formed by the progressive addition of one lauryl residue at a time on a (gallocatechin)_2_ oligomer at 633 Da (with Na+), starting with the (gallocatechin)_2_-laurate (with Na+) oligomer at 815 Da and ending with the (gallocatechin)_2_-(laurate)_9_ (with Na+) at 2271 Da.

The third series is composed of the peaks at 617 Da, 981 Da, 1163 Da, 1344 Da, 1526 Da (shoulder), 1708 Da, 1890 Da, 2072 Da, and 2254 Da. These are formed by the progressive addition of one lauryl residue at a time on a gallocatechin-robinetinidin oligomer at 617 Da (with Na^+^). However, an expected peak at 798 Da that should represent a gallocatechin-robinetinidin-laurate oligomer does not appear in the spectrum. Thus, the series of laurate peaks starts with the 981 Da gallocatechin-robinetinidin-(laurate)_2_ and ends with the 2254 Da gallocatechin-robinetinidin-(laurate)_9_, all with Na+. The peak at 1526 Da is really a barely visible shoulder.

There then exists a fourth series of rather small peaks, namely 587–589 Da, 953 Da, 1317 Da, 1499 Da, 1681 Da (shoulder), 1863 Da, and 2045 Da. These are formed by the progressive addition of one lauryl residue at a time on a fisetinidin-robinetinidin oligomer represented by 585–589 Da peaks (with Na+). The oligomer sequence here starts with the peak at 953 Da, being a fisetinidin-robinetinidin-(laurate)_2_, and ends with the peak at 2045 Da, being fisetinidin-robinetinidin-(laurate)_8_, all with Na+. Two peaks that should be in the series are not present, namely one that should be at 771 Da and the other at 1135 Da. These represent the absent oligomers fisetinidin-robinetinidin-laurate and fisetinidin-robinetinidin-(laurate)_3_, respectively, both absent from the spectra. One can only speculate why this occurs, but it could be that these two species might be particularly reactive, and they are not replenished once a further lauryl chloride reacts with them, hence not surviving to the conditions of esterification of the tannin that have been used.

### 3.3. Density and Friability

**Figure 8:**
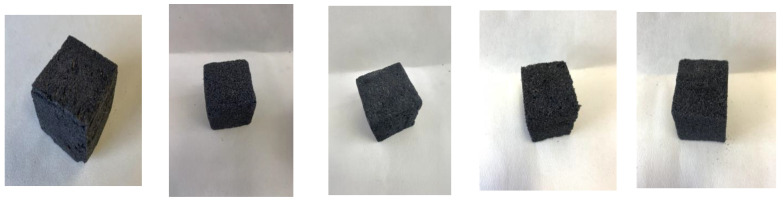
From left to right: examples of cubes of standard foam, Foam A, Foam B, Foam C, and Foam D.

In Table 1 are shown the formulations used to prepare the oil-grafted tannin foams. A small amount of oil-grafted tannin prepared with palmitoyl chloride was added into the formulation to increase the wettability of the foam. Figure 8 shows examples of foam cubes of each formulation listed in Table 1, while the respective densities of the five different formulations in which the additive was palmitoyl-grafted tannin are shown in Table 5. Table 5 shows that the density of the standard foam was higher than the densities of the foams in which a palmitoyl-grafted tannin additive had been included. This is worth keeping in mind when measuring the respective friability of these foams, as in general, for the standard formulation the lower the density the more friable the foam.

**Figure 9:**
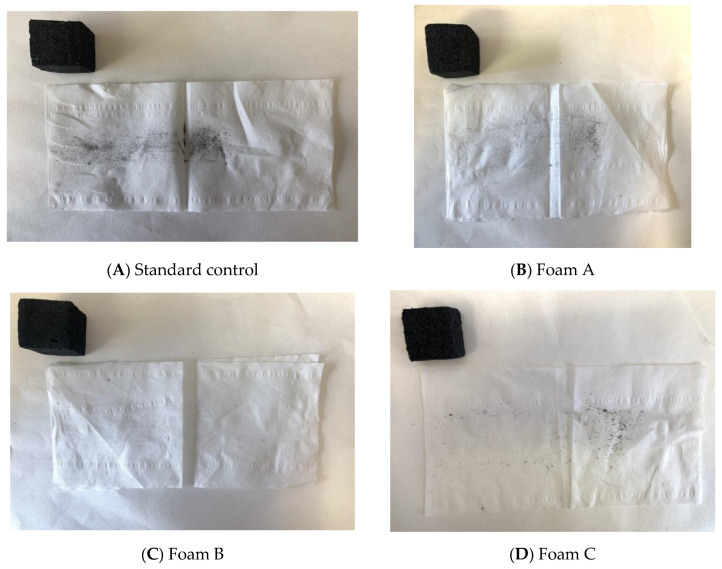

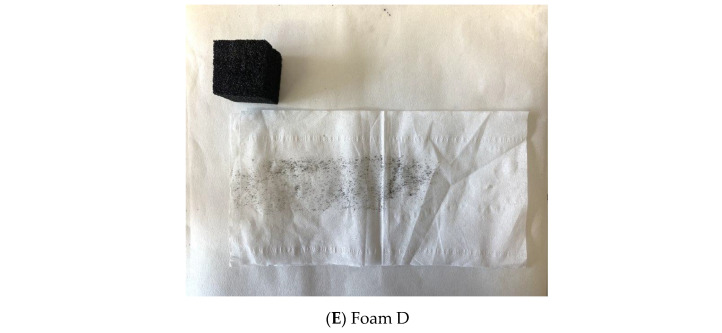
Surface friability test of the standard foam and the four oil-grafted foams A, B, C, and D.

Figure 9 shows the improvement in friability that occurred with the different foams to which the palmitic oil residues grafted to the tannin had been used as an additive in a relatively small amount [23,33]. The figures show, from the black trace of foam material produced by friction on the white paper, that the four experimental formulations are in general markedly less friable than the standard foam, and this at a lower density of the standard foam (Table 5). From more friable to less friable, their order is Standard>D>A>C>>B (Figure 9). Thus, Formulation B is clearly the best of the four experimental foams, with a non-friable surface, a considerable advantage for its application in a number of uses. The main difference from the other foams is the presence of glutaraldehyde as a cross-linker, while the use of acetaldehyde is not as performant in terms of eliminating friability, as can be seen from the friability of foam C that is worse than for foam D.

### 3.4. Wettability

**Figure 10:**
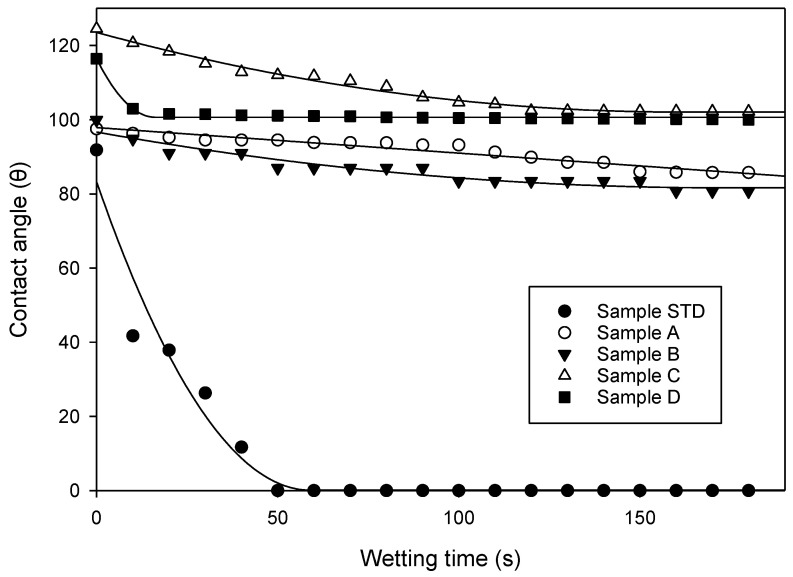
Wetting angle and its variation as a function of time of the (●) standard foam, (◯) foam A, (▼) foam B, (△) foam C, and (■) foam D.

Figure 10 shows the results of the initial contact angle of a sessile water drop on the surface of the five foams, and the variation of the contact angle as a function of time in the first 180 s. The contact angle of the standard foam is high, around 90° at the first second of measure, but it decreases abruptly after 10 s and after 50 s the water droplet has completely penetrated into the foam. This is not the case, however, for the foams with an oil-grafted tannin additive. After 150 s the contact angle reading became static and remained the same for samples A, B, and C. For sample D it became static after just 20 s. This means that adding the oil-grafted tannin additive increases the water-repellent property of the four experimental foams. Thus, the water drop contact angle becomes stable in the order from the best to the worst of D>B=C>A>Standard. The respective surface energies were also calculated (Table 6).

Table 6 shows the total surface free energy (SFE) for all the foams, both the standard one and the four with a palmoyl-grafted tannin additive. The total $\gamma_{s}\;$ value decreases as the contact angle of the surface increases. It can be shown that samples A, B, C, and D are hydrophobic, since the value of the polar SFE component $\gamma_{s}^{p}$ is smaller than that of the value of the dispersive SFE $\gamma_{s}^{d}$, thus proving that the addition of palmitic oil-grafted mimosa tannin to a tannin foam increases its water repellence. This confirms that Foams D and C are the most water-repellent, while A and B are less water-repellent, and the standard foam is not water-repellent, thus confirming the results of the contact angle test.

Table 7 reports the values of γl = total surface free energy of liquid; $\gamma_{l}^{p}$ = polar surface energy component of liquid; $\gamma_{l}^{d}$ = dispersive energy component of liquid. This shows that water is a very polar liquid and supports the water repellence of the foams as discussed above from the results in Table 6.

## 4. Conclusions

Commercial mimosa tannin extract was esterified with palmitic, oleic, or lauric acid by reaction with their respective chlorides, with good yields. When used as a small proportion additive in tannin-furanic foams, the esterified tannins rendered the surface of these foams much less or even not at all friable, and very markedly improves both their water repellence and its maintenance over time in relation to a tannin-furanic foam control without this additive. Chemical analysis indicated that esterification of the tannin occurred and indicated a range of esterified tannin oligomers which were formed. These results indicate that this route is viable for eliminating the two problems of friability and wettability of these biofoams.

## Figures and Tables

**Table 1:** Composition of standard tannin-based foams and tannin-based foams formulated with oil-grafted tannins.

Formulation	STD	A	B	C	D
Mimosa tannin extract (g)	15	13.75	13.75	13.75	13.75
Oil-grafted mimosa tannin (g)	-	1.25	1.25	1.25	1.25
Formaldehyde (g)	3.7	3.7	0	0	0
Furfuryl alcohol (g)	6	5.5	5.5	5.5	6
Water (g)	2	0	0	0	2
Glycerol (g)	0	3	2.5	2.5	0
pTSA (g)	5.5	5.5	6	6	5.5
Diethyl ether (g)	1.7	1.7	1.7	1.7	1.7
Glutaraldehyde (g)	0	0	3.7	0	0
Acetaldehyde (g)	0	0	0	3.7	3.7

**Table 2:** Yield of oil-grafted tannins using different fatty acid chlorides.

Fatty Acid Chloride	Yield of Oil-Grafted Tannin after Drying (g)
Lauryl	7.60
Palmitoyl	7.85
Oleyl	8.36

**Table 3:** Different compositions used to produce oil-grafted tannin using palmitoyl chloride.

Mass Ratio T:P	Palmitoyl Chloride (g)	Chloroform (g)	Tannin (g)
2:1	0.25	30	0.5
1:1	0.5	30	0.5
1:4	2	30	0.5
1:8	4	30	0.5

(T = Tannin, P = Palmitoyl chloride).

**Table 4:** Characterization by MALDI-TOF of oil-grafted tannins using palmitoyl chloride.

Molecular Species	Experimental (Da)	Calculated (Da)	Remarks
Fisetinidin-palmitate, protonated 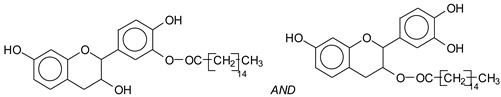	515 Da	513 Da	Free flavonoid monoester
Robinetinidin-palmitate, protonated, no Na^+^ 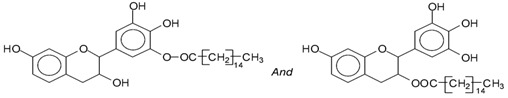	529 Da	528 Da	Free flavonoid monoester, protonated
Gallocatechin-palmitate 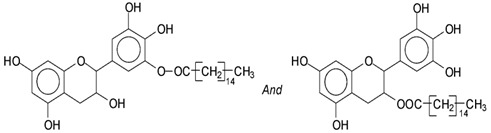	545 Da No Na+	544 Da No Na^+^	Free flavonoid monoester, protonated
Fisetinidin-(palmitate)_2,_ no Na^+^ 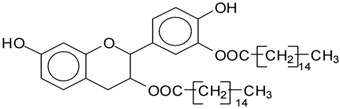	747 Da	750 Da	Free flavonoid diester, deprotonated
(Fisetinidin)_2_-palmitate 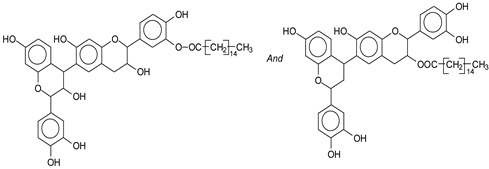	784 Da No Na^+^ 807 Da with Na^+^	784 Da No Na^+^ 807 Da with Na^+^	Flavonoid dimer monoester
(Robinetidin)_2_-palmitate	801 Da	800 Da	Flavonoid dimer monoester, protonated
Gallocatechin-(palmitate)_2_ + Na^+^ 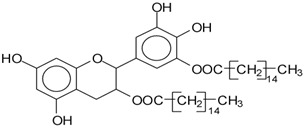	782 Da No Na^+^ 805 Da with Na^+^	782Da No Na^+^ 805 Da with Na^+^	Free flavonoid diester
(Fisetinidin)_2_-(palmitate)_2_ 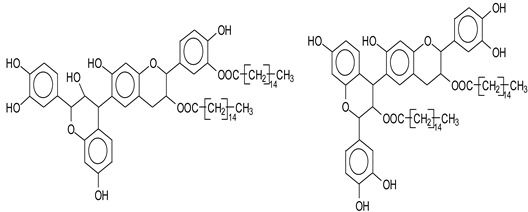	1021Da No Na^+^ 1046 Da with Na^+^	1021Da No Na^+^ 1046 Da with Na^+^	Flavonoid Dimer diester
Robitenidin-(palmitate)_3_	1027 Da with Na^+^	1027 Da with Na^+^	Flavonoid monomer triester
(Fisetinidin)_2_-(palmitate)_3_	1281 Da with Na^+^	1282 Da with Na^+^	Flavonoid Dimer triester
Robinetinidin-fisetinidin-(palmitate)_3_	1297 Da with Na^+^	1298 Da with Na^+^	Dimer triester
(Robinetinidin)_2_-(palmitate)_3_, protonated	1315 Da with Na^+^	1314 Da with Na^+^	Dimer triester
Robinetinidin-Gallocatechin-(palmitate)_3_, protonated	1331 Da with Na^+^	1329 Da with Na^+^	Dimer triester,
Glucose-(fisetinidin)_2_-(palmitate)_3_	1442 Da with Na+	1443 Da with Na^+^	Glucoside dimer triester
Robinetinidin-(fisetinidin)_2_-(palmitate)_3_	1571 Da with Na^+^	1571 Da with Na^+^	Trimer triester
(gallocatechin)_2_-(palmitate)_4_	1580 Da no Na^+^	1580Da no Na^+^	Dimer tetraester
(Robinetinidin)_2_-fisetinidin-(palmitate)_3_	1587 Da with Na^+^	1587 Da with Na^+^	Trimer triester

**Table 5:** Density of tannin foams with oil-grafted tannin.

Foam Sample	Density (g/ $cm^{3})$
Standard foam	0.12
Formulation A	0.0815
Formulation B	0.0853
Formulation C	0.0849
Formulation D	0.1061

**Table 6:** Contact angle measurement and value of total surface free energy (SFE).

Sample	Contact Angle (°)	Surface Energies $\left(mN\;m^{-1}\right)$		
	Water	$\gamma_{s}$	$\gamma_{s}^{d}$	$\gamma_{s}^{p}$
STD	91.83 (19.81)	17.06	8.26	8.8
A	97.51 (3.89)	13.76	7.91	5.85
B	99.93 (5.21)	12.47	6.66	5.81
C	124.54 (7.07)	3.41	3.94	−0.53
D	116.44 (3.66)	5.60	5.05	0.55

where $\gamma_{s}$ = total surface free energy of foam’s solid, $\gamma_{s}^{d}$ = dispersive surface energy of foam’s solid, and $\gamma_{s}^{p}$ = polar surface energy component of foam’s solid.

**Table 7:** Surface energy of liquids with polar and dispersion surface energy components.

Liquid	$\gamma_{l}\left(mN\;m^{-1}\right)$	$\gamma_{l}^{p}\left(mN\;m^{-1}\right)$	$\gamma_{l}^{d}\left(mN\;m^{-1}\right)$
Water	72.8	51	21.8

## Supplementary Materials

The following are available online at https://www.mdpi.com/article/10.3390/polym14225025/s1, Table S1: Characterization of oil-grafted tannins with oleoyl chloride using MALDI-TOF; Figure S1: MALDI-TOF mass spectra of oil-grafted tannin with oleoyl chloride (a) from 400 Da to 800 Da, (b) from 800 Da to 1200Da, (c) from 1200 Da to 1600 Da, (d) from 1600 Da to 2400 Da; Table S2: Characterization of oil-grafted tannin with lauryl chloride using MALDI-TOF; Figure S2. MALDI-TOF mass spectra of oil-grafted tannin with lauryl chloride (a) from 400 Da to 800 Da, (b) from 800 Da to 1200 Da, (c) from 1200 Da to 1600 Da, (d) from 1600 Da to 2000 Da, (e) from 2000 Da to 2500 Da.
