# Supplementary material for: Development of Water Repellent, Non-Friable Tannin-Furanic-Fatty Acids Biofoams
Source: Polymers (Basel). 2022 Nov 19;14(22):5025. doi: 10.3390/polym14225025 (PMC9692386; doi:10.3390/polym14225025)
Supplement: Supplementary file 1 [file polymers-14-05025-s001.zip › polymers-2021844-supplementary.pdf]

SUPPLEMENTARY MATERIAL

Development of Water Repellent, non-Friable Tannin-Furanic-Fatty Acids Biofoams

Elham Azadeh[1], Ummi Hani Abdullah[2,3]*, Nurul Basirah Md Ali[2], Antonio Pizzi[1,4]*, Christine Gerardin-Charbonnier[1], Philippe Gerardin[1], Wan Sarah Samiun[5] and Siti Efliza Ashari[5]

Table S1. Characterization of oil-grafted tannin with oleoyl chloride by MALDI-TOF

Molecules	Peak Experimental	Peak Calculated
Fisetinidin-oleate + Na^+ 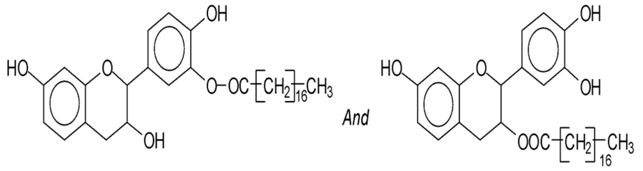	561 Da	561 Da
Robinetinidin-oleate, OR robinetinidin-robinetinidin dimer, no Na+, deprotonated 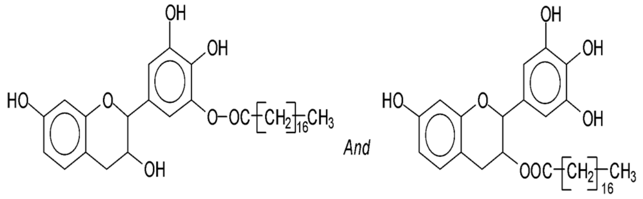	575-577 Da	575-577 Da
Fisetinidin- $(oleate)_2$ no Na^+ 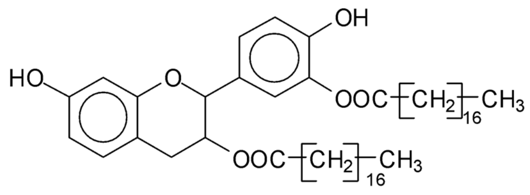	801 Da	801 Da
Robinetinidin- $(oleate)_2$ with Na^+ 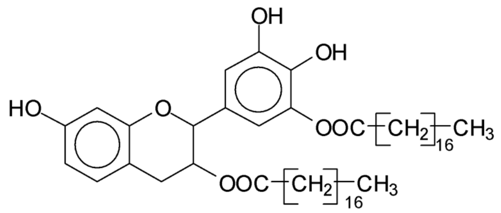	841 Da	841 Da
$(robinetinidin)_2$-gallocatechin trimer, no Na^+	879-881 Da	881 Da
$(gallocatechin)_2$-robinetinidin trimer, no Na^+	895 Da	895 Da
gallocatechin$)_2$-robinetinidin trimer, with Na^+	909 Da	910 Da
Robinetinidin-$(oleate)_3$ with Na^+	1105 Da	1105 Da

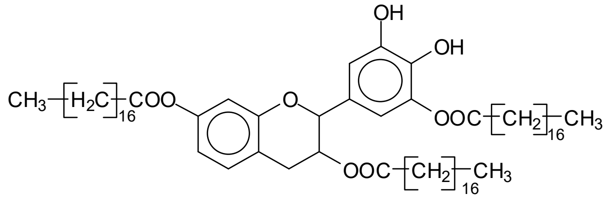		
robinetinidin-gallocatechin–(oleate)$_2$ no Na^+, protonated	1149 Da	1146 Da
fisetinidin-(oleate)$_4$, protonated	1331 Da no Na^+<br>1355 Da with Na^+	1330 Da<br>1354 Da
(fisetinidin)$_2$-(oleate)$_3$ no Na^+<br>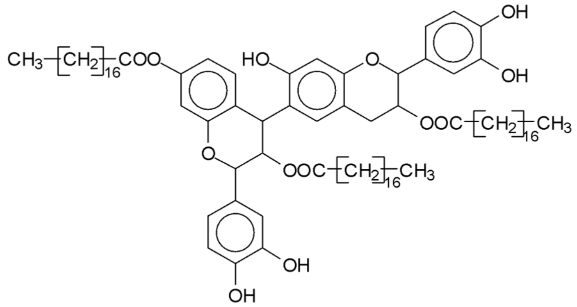	1338 Da	1338 Da
glucose-(fisetinidin)$_2$-(oleate)$_3$,no Na^+	1500 Da no Na+	1500 Da
(fisetinidin)$_2$-(oleate)$_4$	1601 Da no Na+	1601 Da
robinetinidin-(oleate)$_5$, deprotonated	1609 Da no Na+<br>1630 Da with Na^+	1609 Da<br>1631 Da
(gallocatechin)$_2$-(oleate)$_4$, deprotonated	1688 Da with Na^+	1688 Da

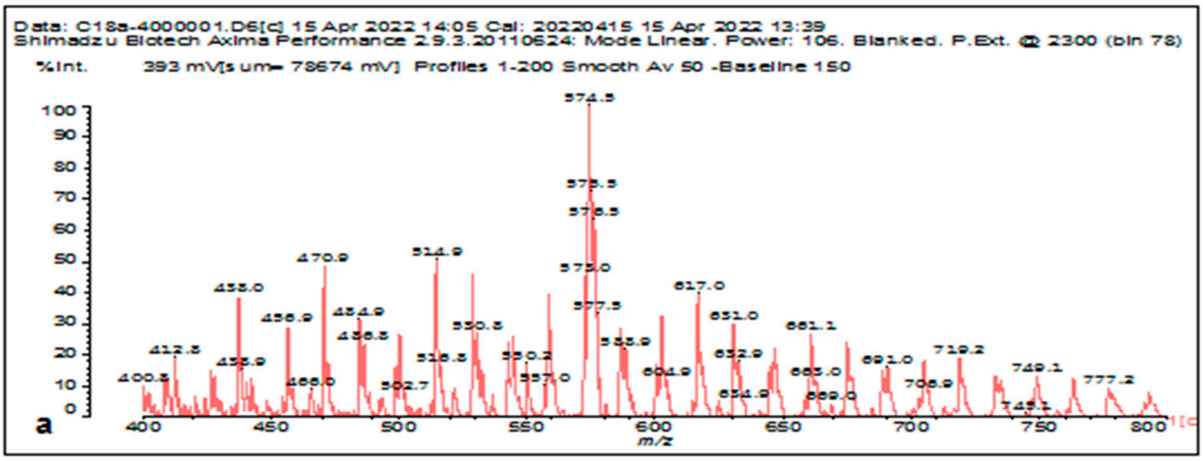

a

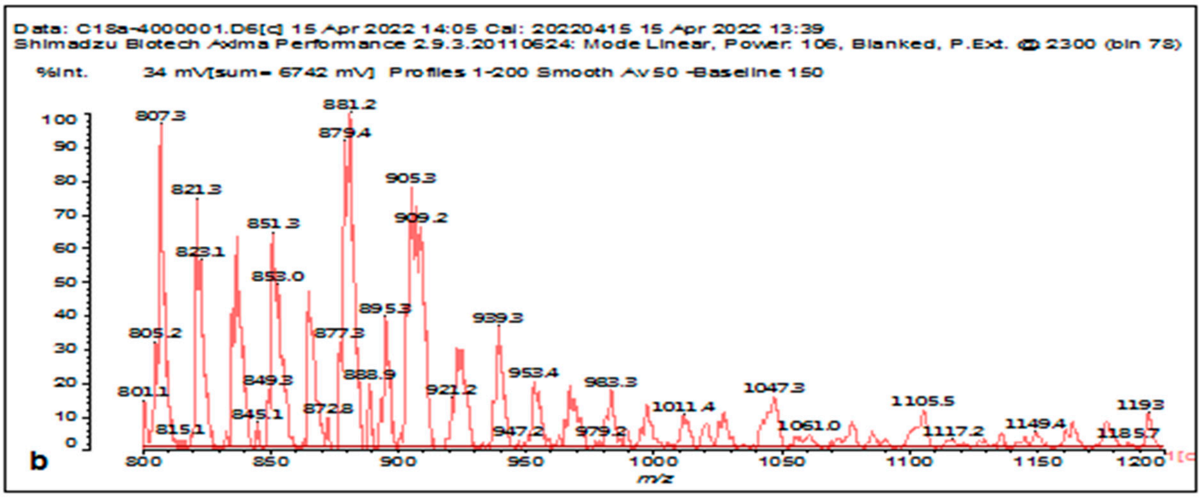

b

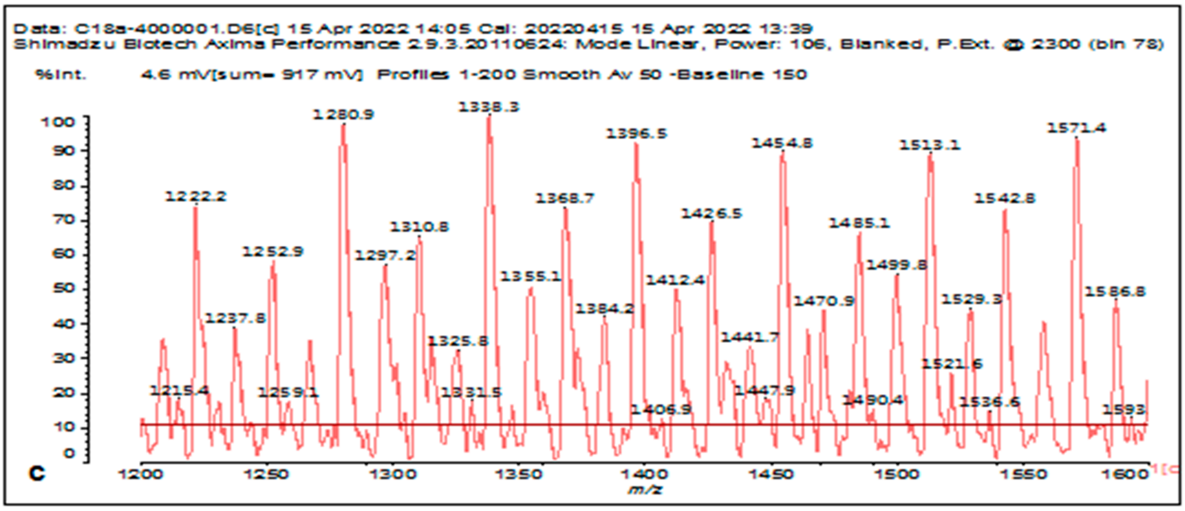

c

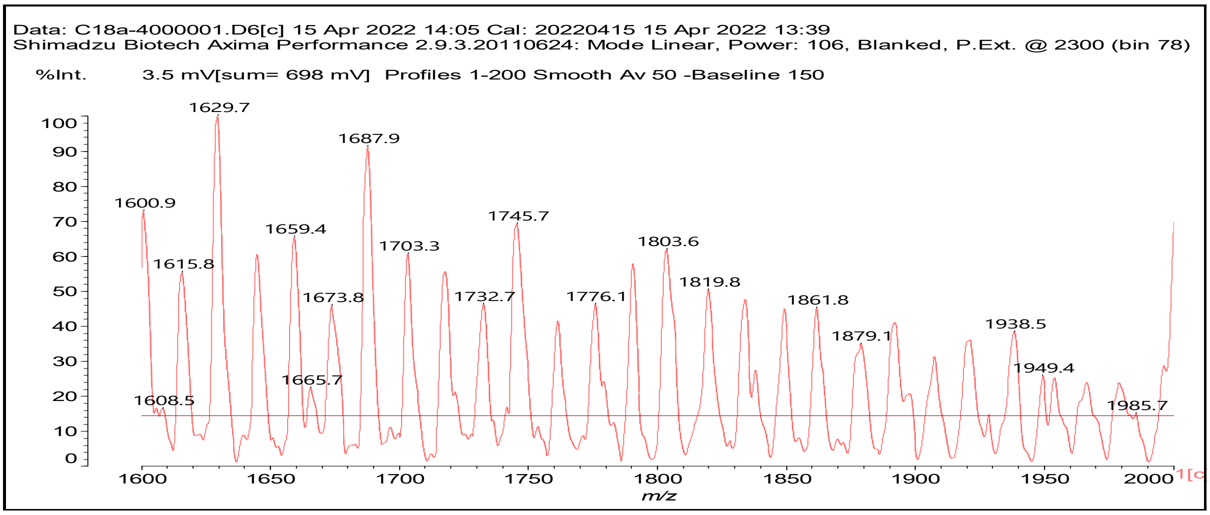

d

Figure S1. MALDI-TOF mass spectrum of oil-grafted tannin with oleoyl chloride (a) from 400 Da to 800 Da, (b) from 800 Da to 1200Da,(c) from 1200 Da to 1600 Da , (d)from 1600 Da to 2400 Da

Table S2. Characterization of oil-grafted tannin with lauryl chloride by MALDI-TOF

Molecules	Peak Experim-ental	Peak Calculated
Fisetinidin-laurate, protonated 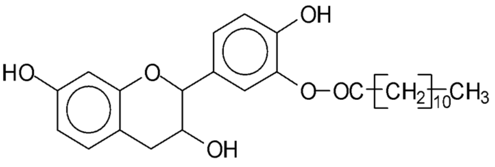 *And* 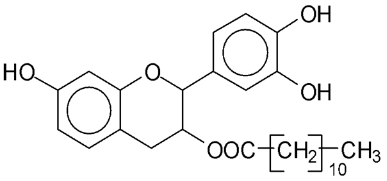	457 Da no Na^+	457 Da
Robinetinidin-laurate 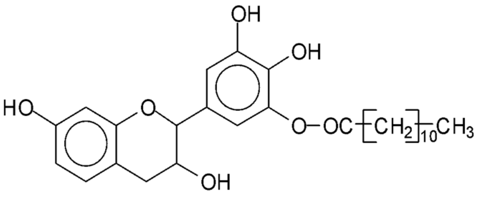 *And* 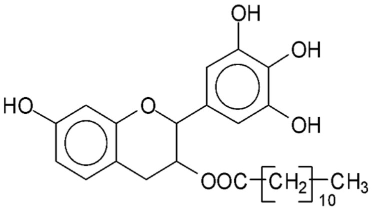	471 Da no Na^+ OK	472 Da
Gallocatechin-laurate 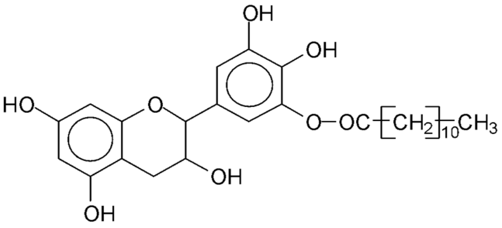 *And* 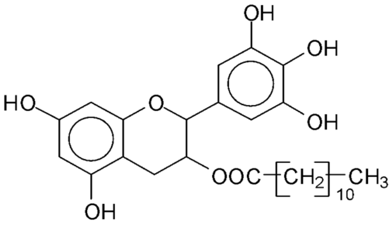	487 Da no Na^+ OK	488 Da
robinetinidin-robinetinidin dimer, deprotonated	575-577 Da no Na^+	576-577 Da
fisetinidin-robinetinidin, protonated	587 Da with Na^+	586 Da
gallocatechin-robinetinidin	617 Da with Na^+	617 Da
(gallocatechin)$_2$	633 Da with Na^+	633 Da
Fisetinidin-(laurate)$_2$ + Na^+ 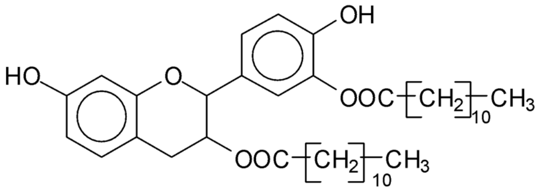	661 Da with Na^+	661 Da
Robinetinidin-(laurate)$_2$ + Na^+ 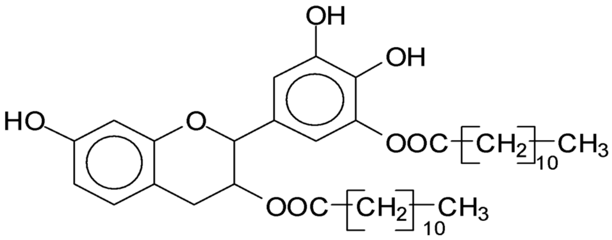	675-677 Da with Na^+ OK	677 Da
Gallocatechin-(laurate)$_2$ + Na^+	691 Da with Na^+ OK	691 Da

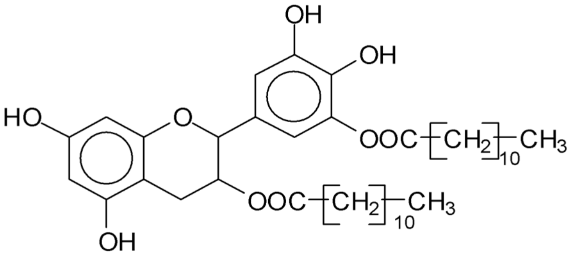		
(fisetinidin)$_2$-laurate<br>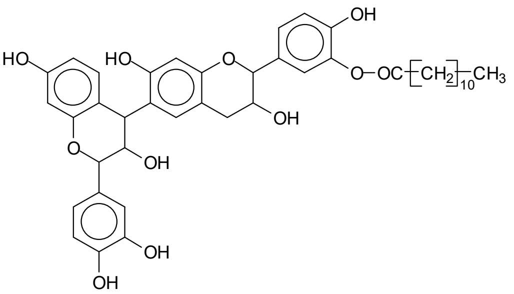 *And* 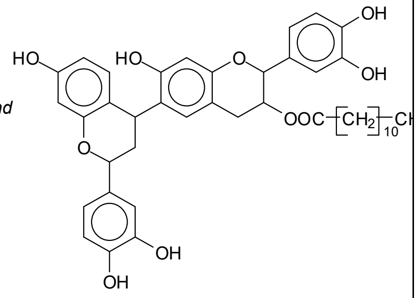	747 Da with Na^+	750
- (robinetinidin)$_2$-laurate, protonated	761 Da no Na^+	760 Da
- robinetinidin-gallocatechin-laurate, protonated	777 Da no Na^+	776 Da
- (gallocatechin)$_2$-laurate	815 Da with Na^+	815 Da
-(robinetinidin)$_2$-gallocatechin trimer, deprotonated	879 Da no Na^+	880 Da
- (gallocatechin)$_2$-robinetinidin trimer, no Na^+, deprotonated	895 Da no Na^+	896 Da
- fisetinidin-robinetinidin-(laurate)$_2$	949-953 Da withNa^+	949 Da
- robinetinidin-gallocatechin–(laurate)$_2$	981 Da with Na^+	981 Da
- (gallocatechin)$_2$-(laurate)$_2$	997 Da with Na^+	997 Da
- glucose-(robinetinidin)$_2$-fisetinidin	1011 Da, no Na^+	1011 Da
- gallocatechin-robinetinidin-(laurate)$_3$	1163 Da with Na^+	1163 Da
- (gallocatechin)$_2$-(laurate)$_3$	1179 Da with Na^+	1179 Da
- Glucose-(robinetinidin)$_2$-fisetinidin-laurate	1193 Da no Na^+	1193 Da
- (fisetinidin)$_2$-robinetinidin-(laurate)$_2$	1221 Da with Na^+	1221 Da
- (robinetinidin)$_2$-fisetinidin-(laurate)$_2$	1237 Da with Na^+	1237 Da
- (robinetinidin)$_3$-(laurate)$_2$ with Na^+	1253 Da with Na^+	1253 Da
- (robinetinidin)$_2$-gallocatechin-(laurate)$_2$	1269 Da with Na^+	1269 Da
- fisetinidin-robinetinidin-(laurate)$_4$	1317 Da with Na^+	1317 Da
- (gallocatechin)$_2$-(laurate)$_{4-}$	1338 Da no Na^+	1338 Da
- (gallocatechin)$_2$-(laurate)$_{4-}$	1361 Da with Na^+	1361 Da
- gallocatechin-robinetinidin-(laurate)$_4$	1344 Da with Na^+	1344 Da
- Glucose-(robinetinidin)$_2$-fisetinidin-(laurate)$_2$	1375 Da no Na^+	1375 Da
- (robinetinidin)$_2$-fisetinidin-(laurate)$_3$	1419 Da with Na^+	1419 Da
- (robinetinidin)$_3$-(laurate)$_3$	1435 Da with Na^+	1435 Da
- (robinetinidin)$_2$-gallocatechin-(laurate)$_3$	1451 Da with Na^+	1451 Da
- robinetinidin-(gallocatechin)$_2$-(laurate)$_3$	1467 Da with Na^+	1467 Da
- fisetinidin-robinetinidin-(laurate)$_5$	1499 Da with Na^+	1499 Da
- robinetnidin-gallocatechin-(laurate)$_5$	1526 Da with Na^+	1526 Da
- (gallocatechin)$_2$-(laurate)$_5$	1543 Da with Na^+	1543 Da
- Glucose-(robinetinidin)$_2$-fisetinidin-(laurate)$_3$	1557 Da no Na^+	1557 Da
- fisetinidin-robinetinidin-(laurate)$_6$	1681 Da with Na^+	1681 Da
- robinetnidin-gallocatechin-(laurate)$_5$	1708 Da with Na^+	1708 Da
- (gallocatechin)$_2$-(laurate)$_6$	1724 Da with Na^+	1724 Da
- Glucose-(robinetinidin)$_2$-fisetinidin-(laurate)$_4$	1740 Da no Na^+	1740 Da
- fisetinidin-robinetinidin-(laurate)$_7$	1863 Da with Na^+	1863 Da

- robinetnidin-gallocatechin-(laurate)$_6$	1890 Da with Na^+	1890 Da
- (gallocatechin)$_2$-(laurate)$_7$	1906 Da with Na^+	1906 Da
- Glucose-(robinetinidin)$_2$-fisetinidin-(laurate)$_5$	1922 Da no Na^+	1922 Da
- Glucose-(robinetinidin)$_2$-fisetinidin-(laurate)$_6$	2014 Da no Na^+	2014 Da
- (robinetinidin)$_2$-(laurate)$_7$, protonated	2036 Da no Na^+	2035 Da
- robinetnidin-gallocatechin-(laurate)$_7$	2057 Da no Na^+	2057 Da
- gallocatechin-robinetinidin-(laurate)$_7$	2072 Da with Na^+	2072 Da
- (gallocatechin)$_2$-(laurate)$_8$, deprotonated	2089 Da with Na^+	2089 Da
- gallocatechin-robinetinidin-(laurate)$_8$	2254 Da with Na^+	2254 Da
- Glucose-(robinetinidin)$_2$-fisetinidin-(laurate)$_7$	2286 Da no Na^+	2286 Da
- (gallocatechin)$_2$-(laurate)$_9$, deprotonated,	2271 Da with Na^+	2073 Da
- Glucose-(robinetinidin)$_2$-fisetinidin-(laurate)$_8$	2468 Da no Na^+	2468 Da

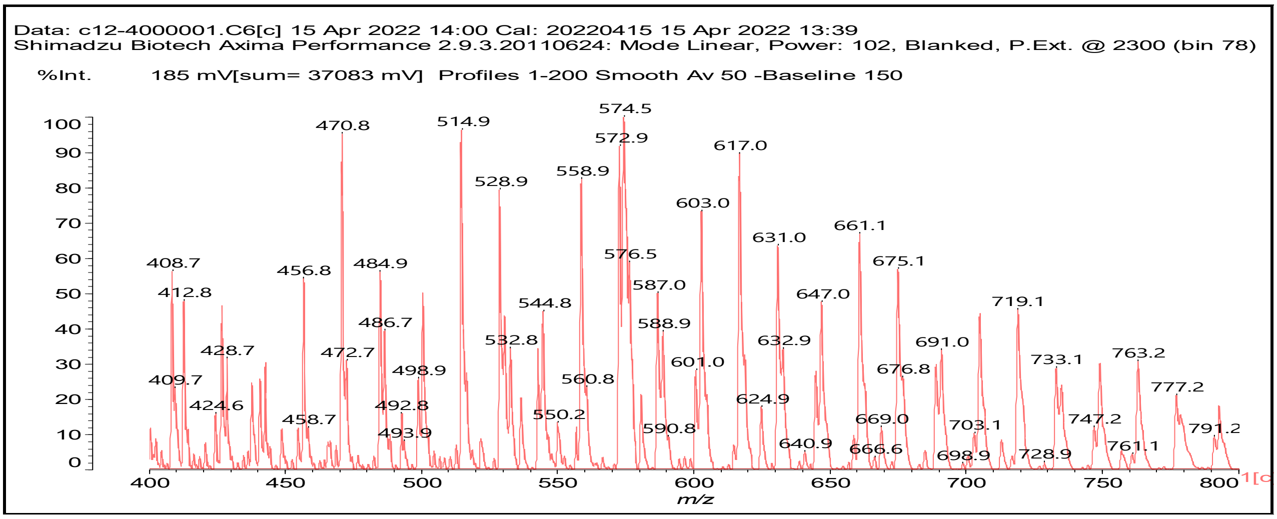

a

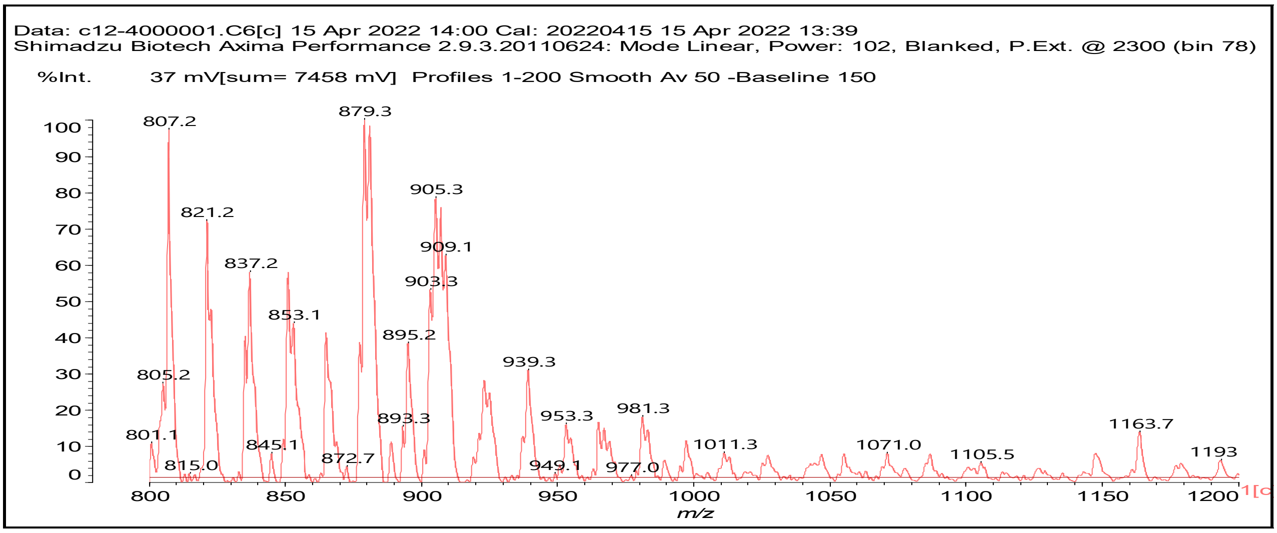

b

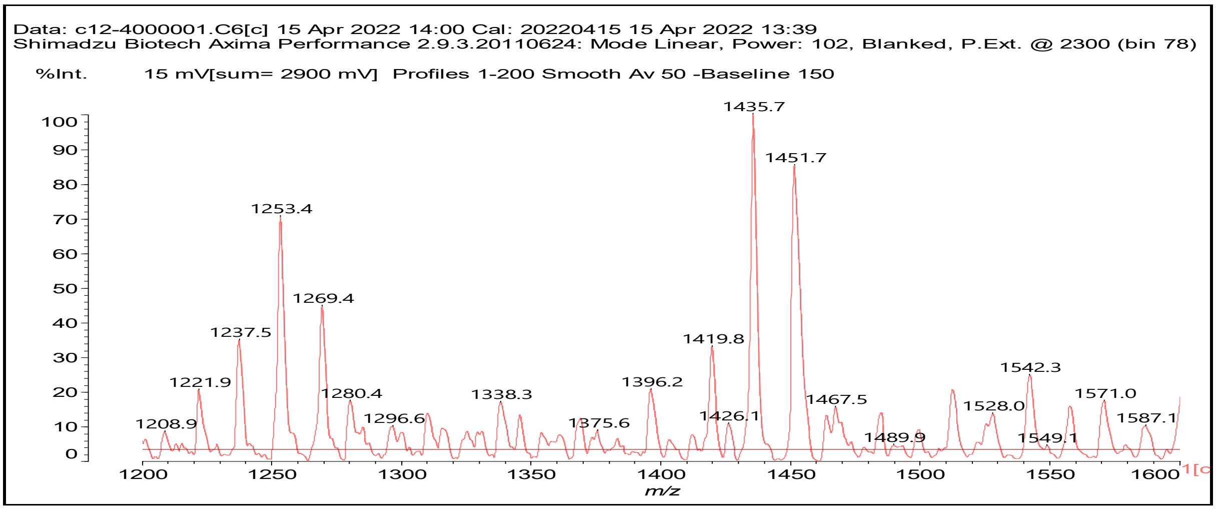

c

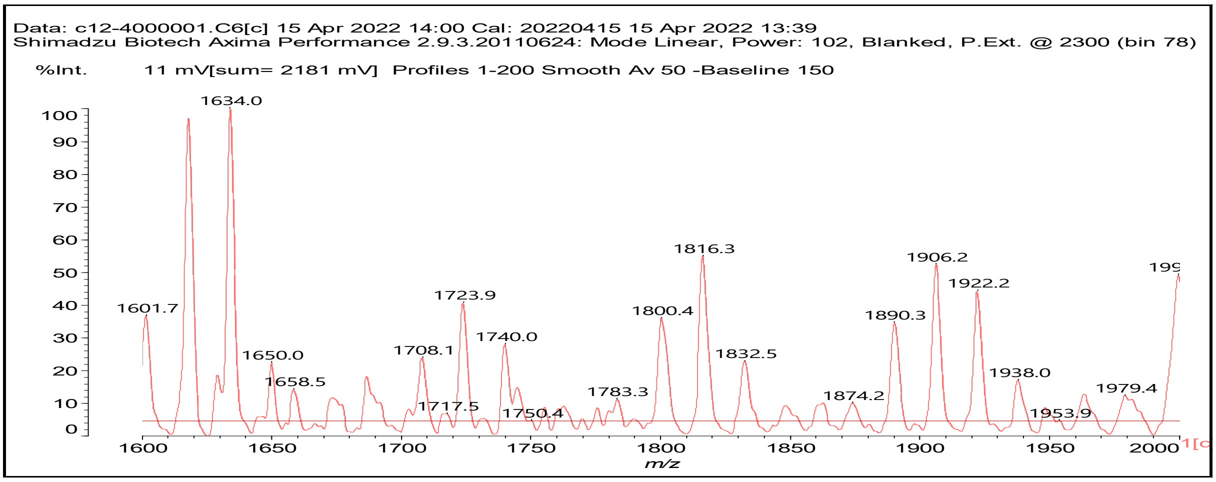

d

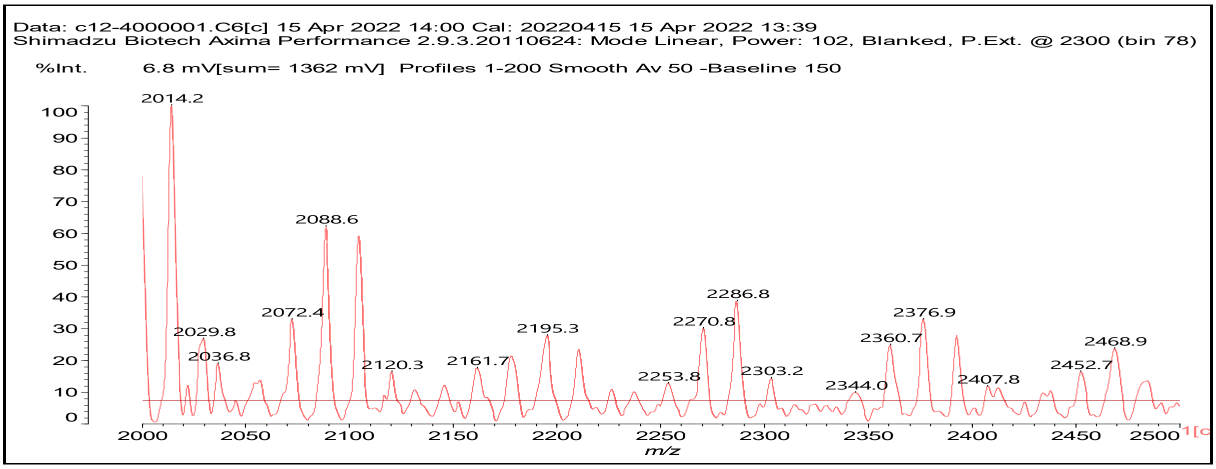

e

Figure S2. MALDI-TOF mass spectrum of oil-grafted tannin with lauryl chloride (a) from 400 Da to 800 Da, (b) from 800 Da to 1200 Da, (c) from 1200 Da to 1600 Da, (d) from 1600 Da to 2000 Da, (e) from 2000 Da to 2500 Da
